# On the Use of Arsenic in Intermittent Fevers

**Published:** 1846-02

**Authors:** 


*On the use of Arsenic in Intermittent Fevers.* By M. Boudin. We find in the Archives Génèrales de Medicine, (September, 1845,) the notice of a paper sent to the Royal Academy of Medicine of Paris, on the use of Arsenic in the treatment of Intermittent Fevers, from which we condense the following facts:

In the course of five years 2947 patients, of all ages, were subjected to the arsenical treatment, without the occurrence of a solitary accident attributable to the remedy. Upwards of 2000 of them had been previously treated from one to ten times by quinine. Nearly 500 of them had been taking quinine, in vain, for several days previously. The cases treated by arsenic were taken promiscuously or without selection, and at all seasons.

The duration of the treatment was usually short, the cases rarely resisting beyond the first or second dose of arsenic. Relapses have been remarkably rare, which may be attributed to the continuance of the remedy eight or ten days after the cessation of the fever. The solutions of Fowler and of Pearson being rather inconvenient to prepare, Mr. Boudin made use of a solution of arsenious acid (white arsenic of the shops) in distilled water. The medium dose of arsenic used was a fifteenth of a grain, given three hours before the expected paroxysm; but if the case seemed obstinate, this dose was preceded by two other similar ones, at intervals of two hours.—*Southern Med. and Surg. Journ.*

